# A redescription of *Syncarpacomposita* (Ascidiacea, Stolidobranchia) with an inference of its phylogenetic position within Styelidae

**DOI:** 10.3897/zookeys.857.32654

**Published:** 2019-06-24

**Authors:** Naohiro Hasegawa, Hiroshi Kajihara

**Affiliations:** 1 Department of Natural History Sciences, Graduate School of Science, Hokkaido University, Kita 10 Nishi 8 Kitaku, Sapporo, Hokkaido 060-0810, Japan Hokkaido University Sapporo Japan; 2 Faculty of Science, Hokkaido University, Kita 10 Nishi 8 Kitaku, Sapporo, Hokkaido 060-0810, Japan Hokkaido University Sapporo Japan

**Keywords:** Chordata, COI, phylogeny, Sea of Okhotsk, taxonomy, Urochordata

## Abstract

Two species of styelid colonial ascidians in the genus *Syncarpa* Redikorzev, 1913 are known from the northwest Pacific. The valid status of the lesser known species, *Syncarpacomposita* (Tokioka, 1951) (type locality: Akkeshi, Japan), is assessed here. To assess the taxonomic identity of *S.composita*, we compared one of the syntypes and freshly collected topotypes of *S.composita* with a syntype of *S.oviformis* Redikorzev, 1913 (type locality: Ul’banskij Bay, Russia). Specimens of *S.composita* consistently differed from the syntype of *S.oviformis* in the number of oral tentacles, the number of size-classes of transverse vessels, and the number of anal lobes. In this paper, *S.composita* is redescribed as distinct from *S.oviformis*, and its phylogenetic position inferred within Styelidae based on the 18S rRNA and cytochrome *c* oxidase subunit I gene sequences. In our phylogenetic tree, *Syncarpa* formed a well-supported clade together with *Dendrodoa* MacLeay, 1824. In *Syncarpa* and *Dendrodoa*, a single gonad is situated on the right side of the body, which is unique among Styelidae, and thus can be a synapomorphy for this clade.

## Introduction

*Syncarpa* Redikorzev, 1913 is a member of the ascidian family Styelidae and consists of two species, *Syncarpacomposita* (Tokioka, 1951) and *S.oviformis* Redikorzev, 1913. The two nominal species *S.corticiformis* Beniaminson, 1975 and *S.longicaudata* Skalkin, 1957, all from the Northwest Pacific, have been synonymized with *S.oviformis* by Sanamyan (2000). This genus is defined by the following four characters: *i)* colonial, with zooids reproducing asexually, *ii*) a single, well-developed fold is present on each side of the pharynx, *iii*) a single gonad is situated on the right side of the body, and *iv*) the gonad has several branches. *Syncarpacomposita* is only known by the original description based on material from Akkeshi, Japan (Tokioka 1951). It was originally placed in a new monotypic genus *Syndendrodoa* Tokioka, 1951, which has been synonymized with *Syncarpa* by Nishikawa (1995).

The phylogeny of ascidians including styelids has been investigated by Zeng et al. (2006), Pérez-Portela et al. (2009), Tsagkogeorga et al. (2009), Alié et al. (2018), and Delsuc et al. (2018). Among these, Alié et al.’s (2018) analysis was based on 4908 genes and included 16 OTUs from Styelidae. It recovered Styelidae as monophyletic with maximum branch-support values, which turned out to be sister to part of paraphyletic Pyuridae. Alié et al.’s (2018) phylogeny showed three major clades for Styelidae: i) Polyzoinae + Botryllinae, ii) *Dendrodoa* + *Polycarpa* + *Polyandrocarpazorritensis* (Van Name, 1931), and iii) *Astrocarpa* + *Styela*. However, no member of *Syncarpa* has ever been placed on a phylogenetic context in any of the previous studies.

The aims of this study are to assess the taxonomic identity of *S.composita* based on type specimens and freshly collected topotypes andto infer the species’ phylogenetic position among Styelidae. In this paper, we redescribe the species and present the results of a multi-gene molecular analysis.

## Materials and methods

Eleven topotype colonies of *S.composita* were freshly collected by dredging, snorkeling, and SCUBA diving in the type locality, Akkeshi Bay, at depths of 3–5 m in June, August, and September 2017, and July 2018 (Table 1). One of the colonies was photographed underwater and in the laboratory with a Nikon COOLPIX AW130 digital camera. The live colonies were anesthetized with menthol; then a part of a zooid was cut off along with the tunic from each colony and preserved in 99% EtOH for DNA extraction. The colonies were preserved in 10% formalin-seawater for morphological observation; zooids were removed from the colonies and then dissected for morphological examination. Larvae for histological observation were dehydrated in an ethanol series, cleared in xylene, embedded in paraffin wax, sectioned at 5 µm thickness, and stained with hematoxylin and eosin. After sections were mounted on glass slides in Entellan New (Merck, Germany), they were observed under an Olympus BX51 compound microscope and photographed with a Nikon D5200 digital camera. These voucher specimens have been deposited in the Invertebrate Collection of the Hokkaido University Museum (ICHUM), Sapporo, Japan. For comparison, specimens deposited in the Seto Marine Biological Laboratory (SMBL), Shirahama, Japan, and the Zoological Institute of the Russian Academy of Sciences (ZIRAS), St. Petersburg, Russia, were also examined.

Total genomic DNA was extracted from a piece of the body wall tissue for eight specimens of *S.composita* as well as one specimen each of *Botrylloidesviolaceus* Oka, 1927, *Pyuramirabilis* (Drasche, 1884), *Styelaclava* Herdman, 1881, and *Styelaplicata* (Lesueur, 1823) (Table 1). The tissue was placed in a 1.5 mL tube after air-dried, then mixed with 180 µL of ATL buffer (Qiagen, Hilden, Germany) and 20 µL of proteinase K (>700 U/mL, Kanto Chemical, Tokyo, Japan), and incubated at 55 °C for ca. 10 h. To the lysis solution, 200 µL of AL buffer (Qiagen) was added and incubated at 70 °C for 10 min; then 210 µL of 99% EtOH was added. The rest of the DNA extraction was carried out following Boom et al.’s (1990) silica method.

**Table 1.** List of specimens newly collected in this study with species, family, sampling date, sampling site, GenBank accession numbers for 18S and COI sequences included in the analysis, and catalog numbers.

Family	Species	Sampling date	Sampling site	GenBank accession number		Catalog number
				18S	COI	
Styelidae	* Botrylloides violaceus *	30 Mar 2017	Oshoro Bay	LC432326	LC432331	ICHUM 5826
	* Styela clava *	26 Au 2017	Shukutsu	LC432329	LC432334	ICHUM 5827
	* Styela plicata *	10 Jul 2017	Moroiso Bay	LC432328	LC432333	ICHUM 5828
	* Syncarpa composita *	25 Jun 2017	Akkeshi Bay	–	–	ICHUM 5815
		25 Jun 2017		–	–	ICHUM 5816
		2 Aug 2017		LC432325	LC432330	ICHUM 5817
		7 Sep 2017		–	–	ICHUM 5818
		7 Sep 2017		–	–	ICHUM 5819
		7 Sep 2017		–	–	ICHUM 5820
		7 Sep 2017		–	–	ICHUM 5821
		7 Sep 2017		–	–	ICHUM 5822
		7 Sep 2017		–	–	ICHUM 5823
		13 Jul 2018		–	–	ICHUM 5824
		13 Jul 2018		–	–	ICHUM 5825
Pyuridae	* Pyura mirabilis *	21 Jun 2017	Oshoro Bay	LC432327	LC432332	ICHUM 5829

Two gene markers were amplified from the genomic DNA by PCR. The nuclear 18S rRNA (18S) gene was amplified with the primer pair 1F/9R (Giribet et al. 1996). The mitochondrial cytochrome *c* oxidase subunit I (COI) gene was amplified with the primers Sty_COI_F2 (5'-TTTGCCTTTAATAGTAAGAAGTCC-3') and Sty_COI_R1 (5'-CATCAAAACAGATGCTGATA-3') for *S.composita* and with the primer pair LCO1490/HCO2198 (Folmer et al. 1994) for the other ascidians. PCRs were performed in a 10-µL total reaction volume with 3 µL of each primer pair (10 µM), 0.5 µL of TaKaRa Ex *Taq* (TaKaRa, Kusatsu, Japan), 10 µL of 10 × Ex *Taq* Buffer (TaKaRa), 8 µL of dNTP mixture (TaKaRa), 1 µL of extracted DNA, and 68.5 µL of deionized water. Thermal cycling condition was 94 °C for 2 min; 35 cycles of 94 °C for 45 sec, 52 °C for 90 sec (for 18S) or 55 °C for 50 sec (for COI), and 72 °C for 55 sec; then 72 °C for 5 min. Amplification was verified by electrophoresis in 1% agarose gel. The PCR products were purified through enzymatic reaction with 24 mU/µL of Exonuclease I (TaKaRa) and 4.9 mU/µL of Shrimp Alkaline Phosphatase (TaKaRa). The purified PCR products were sequenced directly with a BigDye Terminator ver. 3.1 Cycle Sequence Kit (Applied Biosystem, Foster, CA, USA) and 3730 Genetic Analyzer (Applied Biosystems), using the same primer pairs for amplification, as well as the following internal primers for 18S: 3F, 5R (Giribet et al. 1996); and 2, bi (Whiting et al. 1997). Base calling was performed with GeneStudio Professional Edition ver. 2.2.0.0 (GeneStudio, Suwanee, GA, USA).

To infer the phylogenetic position of *S.composita*, 18S and COI sequences of 24 species of Styelidae were obtained from GenBank (Table 2). For 18S, alignment was carried out by MAFFT ver. 7 using the *E-INS-i* strategy (Katoh and Standley 2013); ambiguous sites were removed by using Gblocks ver. 0.91b (Castresana 2002). For COI, nucleotide sequences were manually edited by MEGA ver. 5.2.2 (Tamura et al. 2011) so that translated amino acid sequences were aligned straightforward without indels. 18S and COI sequences were concatenated by using MEGA ver. 5.2.2 (Tamura et al. 2011).

Bayesian inference (BI) was performed using MrBayes ver. 3.2.2 (Huelsenbeck and Ronquist 2001; Ronquist and Huelsenbeck 2003). The best-fit substitution models selected by PartitionFinder ver. 2.1.1 (Lanfear et al. 2016) for BI were GTR+I+G for 18S and GTR+G for all the three codon positions of COI. Each Markov chain was initiated from a random tree and run for 5 × 10^6^ generations; trees were sampled every 100 generation from the chain. Burn-in fraction was set to be 0.25. A consensus of sampled trees was computed using the “sumt” command, and the posterior probability (PP) for each interior branch was obtained to assess the robustness of the inferred relationships. Values of run convergence indicated that sufficient amounts of trees and parameters were sampled (average standard deviation of split frequencies = 0.009823; average estimated sample size of tree lengths = 205.35; potential scale reduction factor of tree lengths = 1.005). Run convergence was also assessed with Tracer ver. 1.6 (Rambaut et al. 2014) to see if the effective sample size of each parameter exceeded 200. Maximum Likelihood (ML) analysis was performed by RAxML ver. 8.2.3 (Stamatakis 2014). One thousand fast-bootstrap replicates were conducted to evaluate nodal support.

**Table 2.** List of species obtained from GenBank included in the phylogenetic analysis with accession numbers for 18S and COI sequences.

Family	Species	GenBank accession number	
		18S	COI
Styelidae	* Botrylloides chevalense *	–	KX650764
	* Botrylloides giganteus *	–	HF922627
	* Botrylloides leachii *	MG009583	KY235402
	* Botrylloides niger *	–	KP254541
	* Botrylloides perspicuus *	–	KY235404
	* Botryllus schlosseri *	FM244858	AY600987
	* Dendrodoa aggregata *	AJ250774	–
	* Dendrodoa grossularia *	L12416	FJ528650
	* Distoma variolosus *	FM897308	FJ528652
	* Eusynstyela hartmeyeri *	FM897309	–
	* Metandrocarpa taylori *	AY903922	–
	* Pelonaia corrugata *	L12440	–
	* Polyandrocarpa anguinea *	–	KY111428
	* Polyandrocarpa misakiensis *	AF165825	–
	* Polyandrocarpa zorritensis *	FM897311	KX138505
	* Polycarpa aurata *	FM897312	FJ528646
	* Polycarpa tenera *	FM897313	FJ528655
	* Polyzoa opuntia *	FM897314	FJ528647
	* Stolonica socialis *	FM897317	–
	* Styela canopus *	–	KU905887
	* Styela gibbsii *	AY903923	HQ916447
	* Styela montereyensis *	L12443	FJ528638
	* Symplegma rubra *	FM897315	FJ528648
	* Symplegma viride *	DQ346655	–
Pyuridae	* Halocynthia roretzi *	AB013016	AB024528

## Systematics

### Family Styelidae Sluiter, 1895

#### Genus *Syncarpa* Redikorzev, 1913

##### Syncarpa composita

Taxon classificationAnimaliaStolidobranchiaStyelidae

(Tokioka, 1951)

Syndendrodoa composita Tokioka, 1951: 14–16, fig. 11.
?Syncarpalongicaudata Skallin, 1957: 297–298, figs a, b.

###### Material examined.

Thirteen specimens: SMBL 104 (syntypes, two colonies); ICHUM 5815–5825 (non-types, each represented by a single colony).

###### Comparative material examined.

ZIRAS 508-911, one of the syntypes of *Syncarpaoviformis* Redikorzev, 1913.

###### Description.

**Figure 1.**
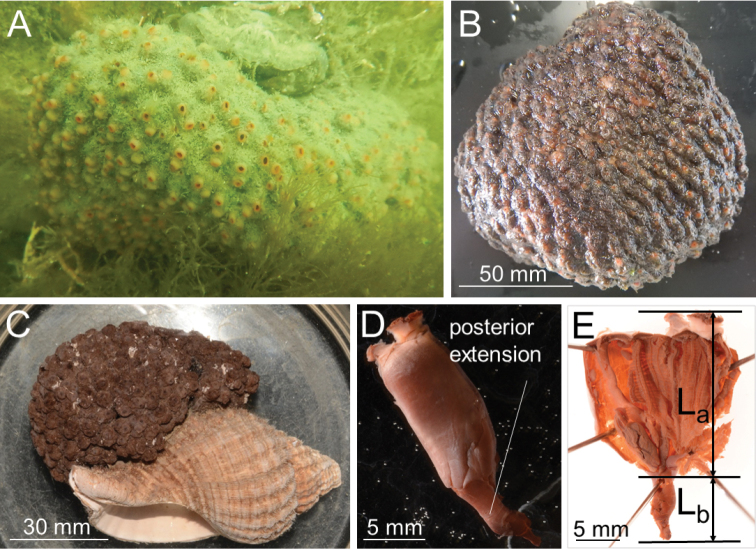
*Syncarpacomposita* (Tokioka, 1951). **A, B, D, E**ICHUM 5817 **C**SMBL 104 (syntype). **A** Live colony **B** intact colony **C** preserved colony **D** intact zooid **E** zooid showing length from top of siphon to end of stomach (L_a_) and from end of stomach to posterior end of zooid (L_b_).

**Figure 2.**
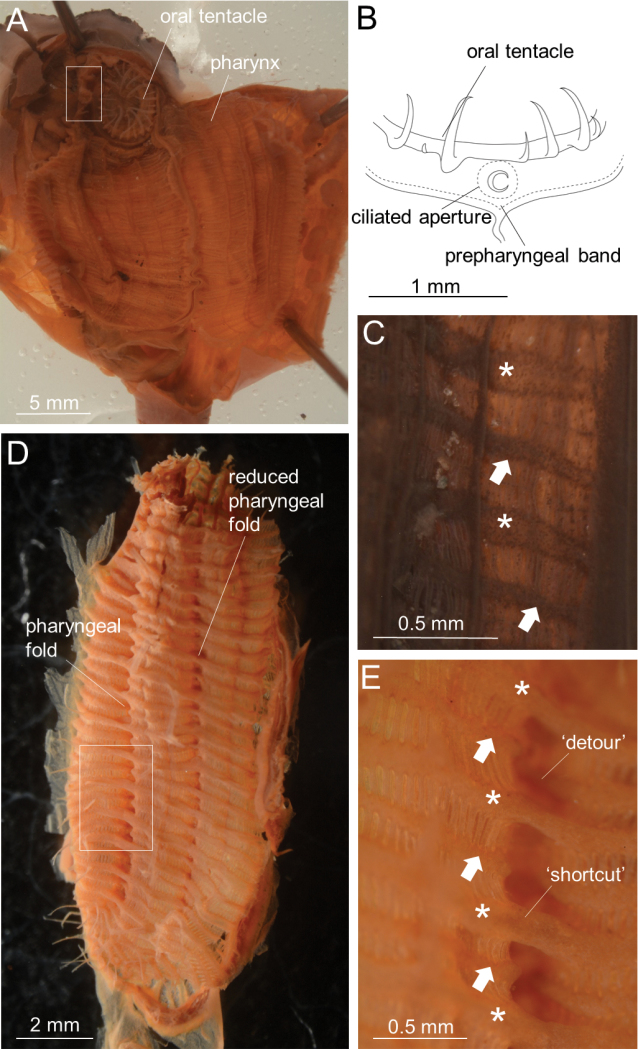
*Syncarpacomposita* (Tokioka, 1951). **A, B, D, E**ICHUM 5817 **C**SMBL 104. **A** Zooid opened dorsally **B** ciliated groove (rotated 90 degrees anti-clockwise and enlarged view of the white square of **A**) **C** magnification of inner surface of pharynx, showing large (indicated by an asterisk) and small (indicated by an arrow) transverse vessels **D** outer surface of pharynx, viewed from right side **E** magnification of white square in **D**, showing ‘shortcut’ of large transverse vessel (asterisk) above pharyngeal fold and ‘detour’ of small transverse vessel (arrowed) along pharyngeal fold.

Colonies ca. 30–50 mm (40 mm and 50 mm in syntypes) in thickness and ca. 40–130 mm (45 mm and 100 mm in syntypes) in diameter. Tunic grayish violet to black or red in life, tough and leathery; zooids more or less protruded and thus externally discernible from each other (Fig. 1A–C). Zooids 12–50 mm long (21 mm and 22 mm in syntypes) and ca. 8 mm wide (Fig. 1D). Posterior extension of zooids varying in length within the colony and among different colonies; while main zooid length (L_a_) varied from 9 mm to 20 mm, posterior extension length (L_b_) varied from 3 mm to 22 mm among 20 zooids from 11 colonies, with L_b_/L_a_ ratio being 0.33–1.83 (Fig. 1E, Table 3). Siphons four-lobed, reddish in life, close together. Approximately 30 oral tentacles present (Fig. 2A), comprised of larger and smaller ones alternating almost regularly. Approximately 30 atrial tentacles present and ca. 0.3 mm long. Ciliated aperture of the dorsal tubercle C-shaped, with its interval directing leftward (Fig. 2B). Prepharyngeal band consisting of a single lamina running close to the ring of oral tentacles; prepharyngeal band V shaped around the dorsal tubercle. Neural ganglion close to dorsal tubercle. Dorsal lamina smoothly margined. One pharyngeal fold and one reduced pharyngeal fold present on each side of pharynx with formula:

**Table 3.** Comparison of the posterior extension length and the ratios of L_a_ to L_b_. Each zooid from two colonies of SMBL 104 was measured.

Catalog number	L_a_ (mm)	L_b_ (mm)	L_b_ / L_a_
ICHUM 5817	9	3	0.33
ICHUM 5821	11	4	0.36
SMBL 104	15	7	0.47
ICHUM 5817	12	6	0.5
ICHUM 5820	14	7	0.5
ICHUM 5819	9	5	0.56
ICHUM 5821	12	7	0.58
SMBL 104	11	7	0.64
ICHUM 5818	14	9	0.64
ICHUM 5825	19	15	0.79
ICHUM 5822	10	8	0.8
ICHUM5819	11	9	0.82
ICHUM 5818	14	12	0.86
ICHUM 5824	22	23	1.05
ICHUM 5823	13	14	1.08
ICHUM 5825	19	25	1.32
ICHUM 5824	20	30	1.5
ICHUM 5823	13	20	1.54
ICHUM 5820	18	29	1.61
ICHUM 5822	12	22	1.83

L D. 0 (7–8) 2 (2) 3 V.

R D. 0 (7) 2 (3) 3 V.

**Figure 3.**
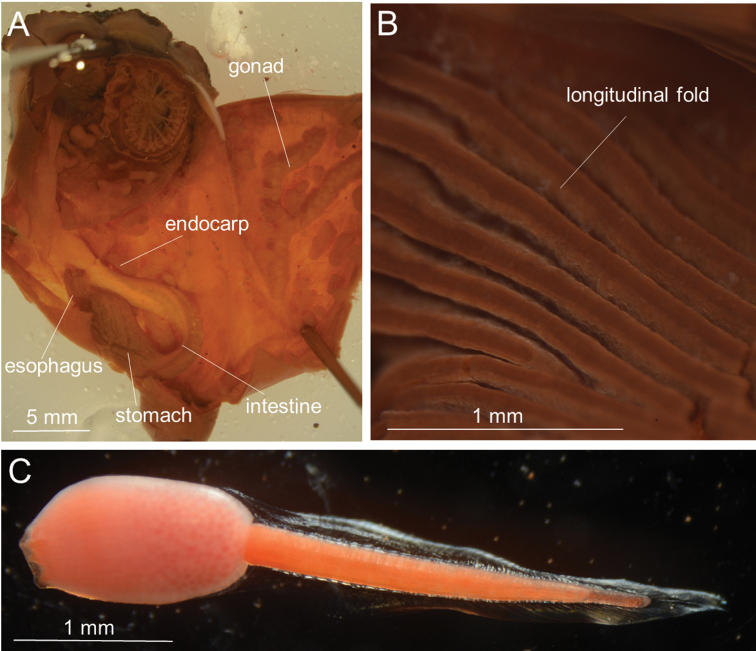
*Syncarpacomposita* (Tokioka, 1951). **A, B**ICHUM 5817 **C**ICHUM 5824. **A** Zooid opened dorsally, with pharynx removed **B** stomach internal surface **C** tadpole larva.

Thirteen-twenty stigmata per mesh between endostyle and first longitudinal vessel from endostyle. Transverse vessels comprised of larger and smaller ones almost regularly alternating antero-posteriorly (Fig. 2C); when running across each pharyngeal fold (as well as reduced pharyngeal fold) on outer surface of pharynx, larger ones always taking a ‘shortcut’ and bridging over fold valley, while smaller ones ‘detour’ and go along valley (Fig. 2D, E). Parastigmatic vessels present. Stigmata straight. Gut located on left side (Fig. 3A). Alimentary system occupying approx. half of the left side of body; intestinal loop J-shaped. Esophagus short and slightly curved; its length being one-third of stomach (Fig. 3A). Stomach spindle-shaped, shorter than one-third of body length and has no plication or striation on its outer surface; stomach lying almost parallel to longitudinal axis of body (Fig. 3A), with its internal wall having at least 22 well-defined, regularly arranged, parallel, longitudinal folds (Fig. 3B). Intestine gently curving from pyloric part. Anus lying almost beneath atrial aperture. Diameter of intestine almost uniform from pylorus to anus. Anus without lobes. Gonad with 2–5 branches, situated only on right side of body (Fig. 3A). Ovaries spherical, occupying medial side of gonad; oviduct slightly bending at its end to peripharyngeal cavity before opening on right side of body at almost same level as pylorus. Male follicles located laterally within gonad, surrounding ovaries. Many endocarps present on inner surface of body wall (Fig. 3A).

**Figure 4.**
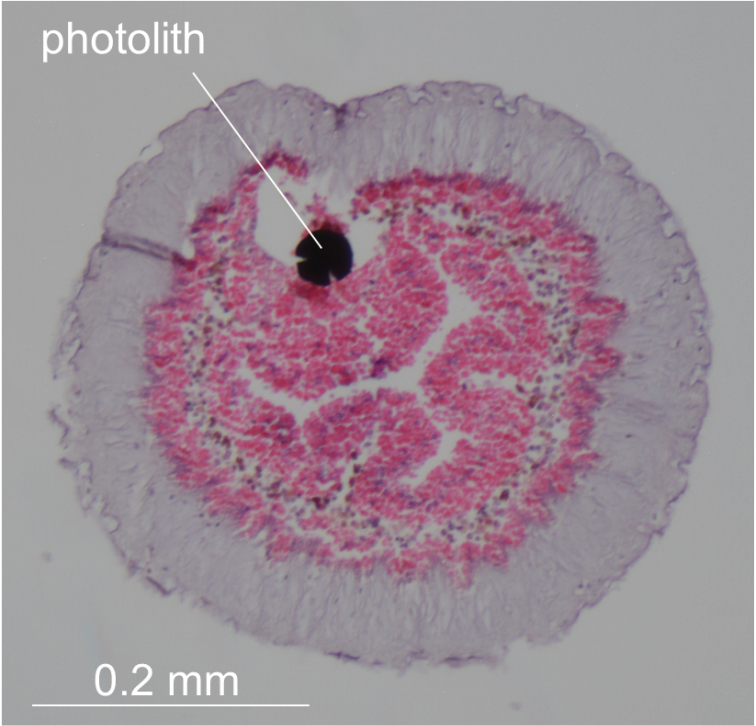
*Syncarpacomposita* (Tokioka, 1951), ICHUM 5824, cross section of a tadpole larva, showing photolith.

Hatched tadpole larvae found in peripharyngeal cavity of ICHUM 5824 and 5825; trunk spindle-shaped, ca. 1 mm in length (Fig. 3C). Three adhesive papillae arranged in triangle. Approximately 35 elongated ampullae discerned on anterior half of trunk surface. Photolith present in cerebral vesicle but invisible from the outside (Fig. 4). Tail twice as long as trunk.

###### Remarks.

**Figure 5.**
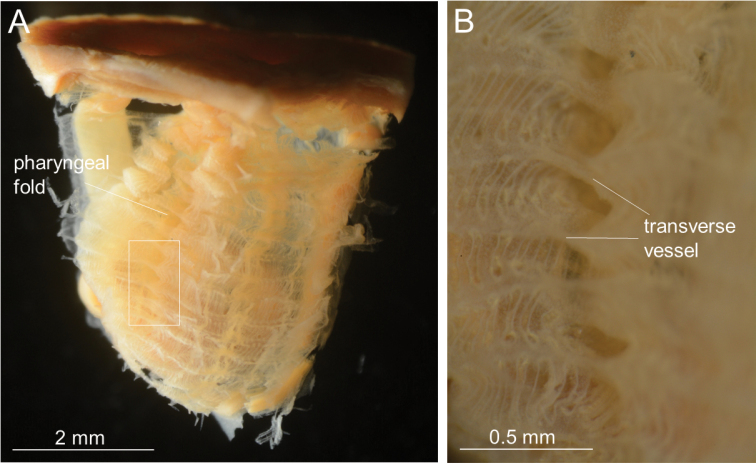
*Syncarpaoviformis* Redikorzev, 1913, ZIRAS 508-911 (syntype). **A** Outer surface of pharynx, viewed from right side **B** magnification of white square in **A**, showing that all transverse vessels make ‘shortcuts’ and bridge across the pharyngeal fold.

*Syncarpacomposita* and *S.oviformis* are different in terms of the number of oral tentacles, the number of size-classes of transverse vessels, and the number of anal lobes (Table 4). In addition, the transverse vessels in *S.composita* alternate ‘shortcut’ and ‘detour’ when crossing the valley of pharyngeal folds, while all the transverse vessels in *S.oviformis* make a shortcut and bridge over the valley of pharyngeal folds (Fig. 5A, B). Based on the consistent, discontinuous differences discovered in the present study, we conclude to leave *S.composita* as a valid species as opposed to *S.oviformis*, until molecular data settle the issue of conspecificity.

*Syncarpacomposita* and *S.longicaudata* were supposed to be differentiated by the ratio of the lengths of the zooid’s main body (L_a_) to its posterior extension (L_b_), expressed as L_b_/L_a_ (Fig. 1E). The values of this character for *S.composita* and *S.longicaudata*, based on the original figures (Tokioka 1951, figs 11.2, 11.3; Skalkin 1957, fig. a), are 0.40 and 1.00, respectively. In this study, however, we discovered that the L_b_/L_a_ values could vary from 0.33 to 1.83 even intra-colonially in *S.composita* (Table 3), completely encompassing the character state of *S.longicaudata*. Although *S.longcaudata* has been considered a junior synonym of *S.oviformis*, we think that it is more similar to *S.composita* (Table 4). Extensive population genetic studies on potentially different populations of these species from the Northwest Pacific would help to improve our understanding of the taxonomy of this genus.

**Table 4.** Comparison of four species of *Syncarpa*. The number of size-classes of transverse vessels in *S.oviformis* (indicated by an asterisk*) was newly confirmed in this study. Sanamyan (2000) concluded that *S.corticiformis* and *S.longicaudata* were junior synonyms of *S.oviformis*.

Character	Species					
	* S. composita *		* S. corticiformis *	* S. longicaudata *	* S. oviformis *	
Source	Tokioka (1951)	present study	Beniaminson (1975)	Skalkin (1957)	Redikorzev (1913)	Sanamyan (2000)
Zooid length (mm)	12	12–50	15	40	10	10–30
Zooid width (mm)	8	8	5	7.5	4	4–8
Posterior extension of zooid long (+) or short (‒)	‒	‒ / +	‒	+	‒	‒
Number of oral tentacles	30	30–35	20	30–35	20–25	20–25
Number of size-classes of transverse vessels	?	2	1	2	1*	?
Stomach internal wall present (+) or absent (‒)	?	+	+	+	+	+
Intestinal loop	?	J-shaped	J-shaped	J-shaped	J-shaped	J-shaped
Number of anal lobes	0	0	2	0	2	2
Number of gonadal branches	2–5	2–5	4	3	2	2–4
Locality	Akkeshi Bay	Akkeshi Bay	Kunashiri Island	South Kuril Islands	Ul’banskij Bay	Sea of Okhotsk

###### Phylogeny.

**Figure 6.**
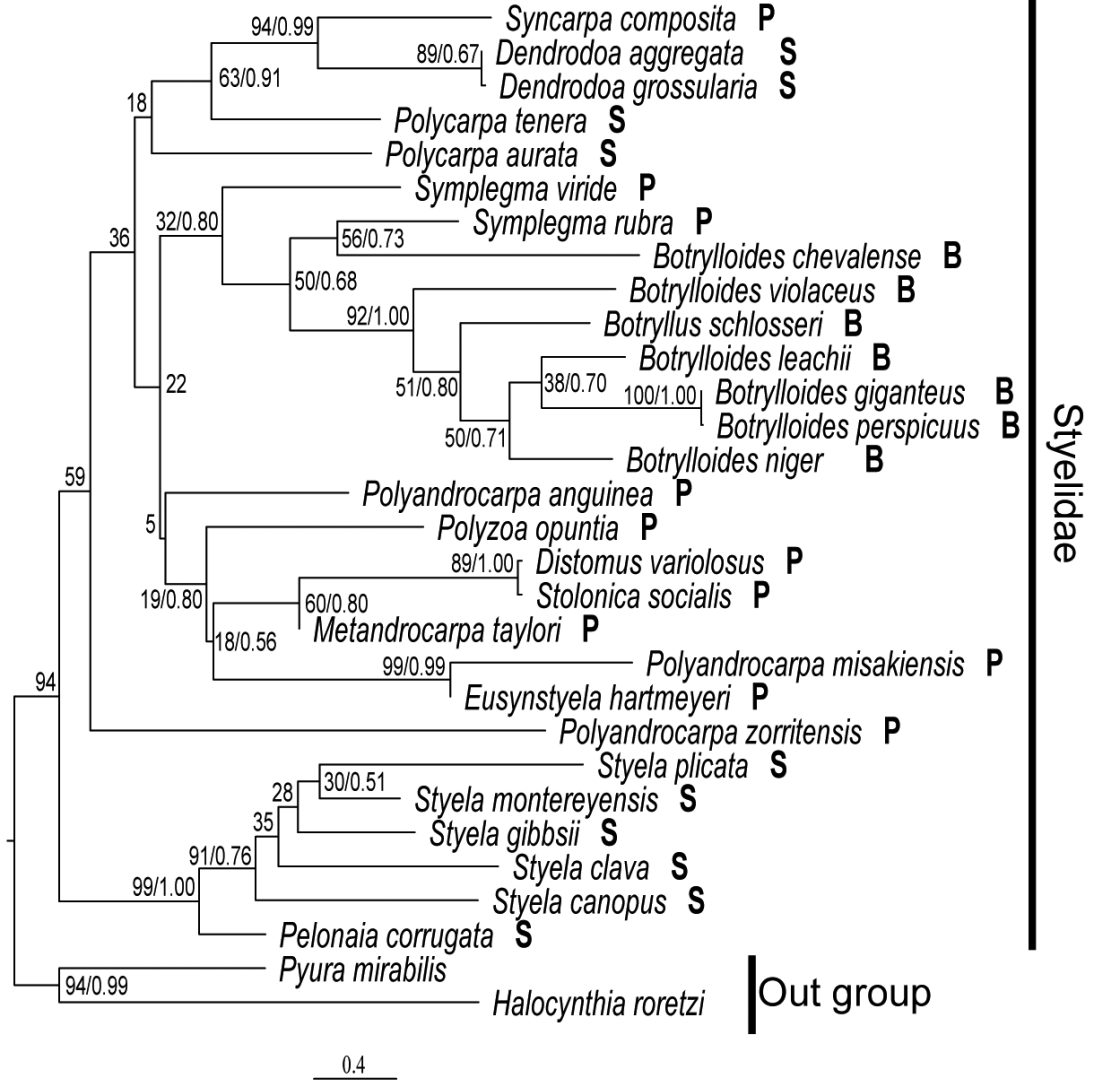
Phylogenetic relationship of 28 styelid ascidians. ML tree generated from concatenated sequences of 18S (1582 bp) and COI (686 bp). Numbers on nodes indicate bootstrap values and, where applicable, posterior probabilities. Scale bar indicates number of substitutions per site. B, P, and S represented Botryllinae, Polyzoinae, and Styelinae.

In the phylogenetic tree, *Syncarpa* formed a well-supported clade together with *Dendrodoa* (Fig. 6). These two genera have a single gonad positioned on the right side of the body. This feature is likely to represent a synapomorphy for this clade. The only difference between *Syncarpa* and *Dendrodoa* is that the former is colonial while the latter is solitary. The latter currently consists of eight species (Shenkar et al. 2019). Future studies should ascertain the possible reciprocal monophyly of the two genera by analyses with expanded taxon sampling from *Dendrodoa*. If they turn out to be reciprocally non-monophyletic (e.g., *Syncarpa* completely nested within paraphyletic *Dendrodoa*), these two genera can be synonymized so that it consists of both colonial and non-colonial species, just as the diazonid *Rhopalaea* Philippi, 1843.

A clade comprised of *Dendrodoa*, *Polycarpa*, and *Polyandrocarpazorritensis* was recovered in Alié et al.’s (2018) phylogenomic analysis based on 4,908 genes, in which *Polyandrocarpazorritensis* was sister to *Polycarpaaurata*, forming a clade sister to *Dendrodoagrossularia*.

Although the nodal support values were generally poor, our tree does not support the three-subfamily classification system: Styelinae consisting of solitary styelid species, Polyzoinae of colonial styelid species without system, and Botryllinae of colonial styelid species with system. Highly reliable molecular analyses and detailed morphological observations including *Syncarpa* would help understanding the systematics of Styelidae.

## Supplementary Material

XML Treatment for Syncarpa composita

## References

[B1] Alié A Hiebert LS Simion P Scelzo M Prünster MM Lotito S Delsuc F Douzery EJP Dantec C Lemaire P Darras S Kawamura K Brown FD Tiozzo S (2018) Convergent acquisition of nonembryonic development in styelid ascidians. Molecular Biology and Evolution 35(7): 1728–1743. 10.1093/molbev/msy068 29660002 PMC5995219

[B2] Beniaminson TS (1975) Morphology and taxonomic position of ascidians of the genus *Syncarpa* Redikorzev with a description of *Syncarpacorticiformis* sp. n. Russian Journal of Marine Biology 3: 29–36.

[B3] Boom R Sol C Beld M Weel J Goudsmit J Dillen PW (1990) Improved silica-guanidiniumthiocyanate DNA isolation procedure based on selective binding of bovine alpha-casein to silica particles. Journal of Clinical Microbiology 1999: 615–619. 10.1128/jcm.37.3.615-619.1999 PMC84491 9986822

[B4] Castresana J (2002) Estimation of genetic distances from human and mouse introns. Genome Biology 3(6): 1–7. 10.1186/gb-2002-3-6-research0028 PMC116725 12093375

[B5] Delsuc F Philippe H Tsagkogeorga G Simion P Tilak M Turon X López-Legentil S Piette J Lemaire P Douzery EJP (2018) A phylogenomic framework and timescale for comparative studies of tunicates. BMC Biology 16: 39. 10.1186/s12915-018-0499-2 PMC5899321 29653534

[B6] Folmer O Black M Hoeh W Lutz R Vrijenhoek R (1994) DNA primers for ampliﬁcation of mitochondrial cytochrome c oxidase subunit I from diverse metazoan invertebrates. Molecular Marine Biology and Biotechnology 3: 294–299. 7881515

[B7] Giribet G Carranza S Baguña J Riutort M Ribera C (1996) First molecular evidence for the existence of a Tardigrada + Arthropoda clade. Society for Molecular Biology and Evolution 13(1): 76–84. 10.1093/oxfordjournals.molbev.a025573 8583909

[B8] Huelsenbeck JP Ronquist F (2001) MRBAYES: Bayesian inference of phylogenetic trees. Bioinformatics Applications Note 17(8): 754–755. 10.1093/bioinformatics/17.8.754 11524383

[B9] Katoh K Standley DM (2013) MAFFT multiple sequence alignment software version 7: improvements in performance and usability. Molecular Biology and Evolution 30(4): 772–780. 10.1093/molbev/mst010 23329690 PMC3603318

[B10] Kott P (1985) The Australian Ascidiacea. Part 1: Phlebobranchia and Stolidobranchia. Memoirs of the Queensland Museum 23: 1–440.

[B11] Lanfear R Frandsen PB Wright AM Senfeld T Calcott B (2016) PartitionFinder 2: new methods for selecting partitioned models of evolution for molecular and morphological phylogenetic analyses. Molecular Biology and Evolution 34(3): 772–773. 10.1093/molbev/msw260 28013191

[B12] MacLeay WS (1824) Anatomical observations on the natural group of Tunicata, with the description of three species collected in Fox Channel during the late northern expedition. Transactions of the Linnean Society of London 14(3): 527–555. 10.1111/j.1095-8339.1823.tb00101.x

[B13] Nishikawa T (1995) Subphylum Urochordata. In: Nishimura S (Ed.) Guide to Seashore Animals of Japan with Color Pictures and Keys, Volume 2.Hoiku-sha, Osaka, 573–608.

[B14] Pérez-Portela R Bishop JDD Davis AR Turon X (2009) Phylogeny of the families Pyuridae and Styelidae (Stolidobranchiata, Ascidiacea) inferred from mitochondrial and nuclear DNA sequences. Molecular Phylogenetics and Evolution 50: 560–570. 10.1016/j.ympev.2008.11.014 19059353

[B15] Philippi A (1843) Rhopalaea ein neues Genus der einfachen Ascidien. Archiv für Anatomie, Physiologie und Wissenschatliche Medicin, 45–57.

[B16] Rambaut A Suchard MA Xie D Drummond AJ (2014) Tracer v1.6. http://beast.bio.ed.ac.uk/Tracer

[B17] Redikorzev V (1913) Neue Ascidien. Zoologischer Anzeiger 43: 204–213.

[B18] Redikorzev V (1941) Ascidien der Meere des fernen Osten der Ud.S.S.R. Investigations of the Far Eastern Seas of the USSR 1: 164–212.

[B19] Ronquist F Huelsenbeck JP (2003) MrBayes 3: Bayesian phylogenetic inference under mixed models. Bioinformatics Applications Note 19(12): 1572–1574. 10.1093/bioinformatics/btg180 12912839

[B20] Sanamyan K (2000) Ascidians from the north-western pacific region 7. Styelidae. Ophelia 53(1): 67–78. 10.1080/00785326.2000.10409436

[B21] Shenkar N Gittenberger A Lambert G Rius M Moreira da Rocha R Swalla BJ Turon X (2019) . Ascidiacea World Database. *Dendrodoa* MacLeay, 1824. Accessed through: World Register of Marine Species. http://www.marinespecies.org/aphia.php?p=taxdetails&id=103531 [on 16 April 2019]

[B22] Skalkin VA (1957) A new species of ascidian from the Pacific Ocean – *Syncarpalongicaudata* sp. n. (family Styelidae). Zoologicheskii Zhurnal 36: 297–298.

[B23] Sluiter CP (1895) Tunicaten. In: Semon R (Ed.) Zoologische Forschungsreisen in Australien und den malayischen Archipel.Denkschriften der Medicinisch-Naturwissenschaftlichen Gesellschaft zu Jena 8: 163–186.

[B24] Stamatakis A (2014) RAxML version 8: a tool for phylogenetic analysis and post-analysis of large phylogenies. Bioinformatics 30: 1312–1313. 10.1093/bioinformatics/btu033 24451623 PMC3998144

[B25] Tamura K Peterson D Peterson N Stecher G Nei M Kumar S (2011) MEGA5: molecular evolutionary genetics analysis using maximum likelihood, evolutionary distance, and maximum parsimony methods. Molecular Biology and Evolution 28(10): 2731–2739. 10.1093/molbev/msr121 21546353 PMC3203626

[B26] Tokioka T (1951) The fauna of Akkeshi Bay XVIII. Ascidia. Publications from the Akkeshi Marine Biological Station 1: 1–22. [2 pls]

[B27] Tokioka T (1963) Contributions to Japanese ascidian fauna. XX. The outline of Japanese ascidian fauna as compared with that of the Pacific Coasts of North America. Publications of the Seto Marine Biological Laboratory 11(1): 131–156. 10.5134/175319

[B28] Tsagkogeorga G Turon X Hopcroft RR Tilak M Feldstein T Shenkar N Loya Y Huchon D Douzery EJP Delsuc F (2009) An updated 18S rRNA phylogeny of tunicates based on mixture and secondary structure models. BMC Evolutionary Biology 9: 187. 10.1186/1471-2148-9-187 PMC2739199 19656395

[B29] Van Name WG (1931) New North and South American ascidians. Bulletin of the American Museum of Natural History 61: 207–255.

[B30] Whiting MF Carpenter JC Wheeler QD Wheeler WC (1997) The Strepsiptera problem: phylogeny of the holometabolous insect orders inferred from 18S and 28S ribosomal DNA sequences and morphology. Systematic Biology 46(1): 1–68. 10.1093/sysbio/46.1.1 11975347

[B31] Zeng L Jacobs MW Swalla BJ (2006) Coloniality has evolved once in stolidobranch ascidians. Integrative and Comparative Biology 46(3): 255–268. 10.1093/icb/icj035 21672740

